# A multimodal vision transformer for interpretable fusion of functional and structural neuroimaging data

**DOI:** 10.1002/hbm.26783

**Published:** 2024-11-26

**Authors:** Yuda Bi, Anees Abrol, Zening Fu, Vince D. Calhoun

**Affiliations:** ^1^ Tri‐institutional Center for Translational Research in Neuroimaging and Data Science (TReNDS), Georgia Tech, Emory Atlanta Georgia USA

**Keywords:** data fusion, neuroimaging, vision transformer

## Abstract

Multimodal neuroimaging is an emerging field that leverages multiple sources of information to diagnose specific brain disorders, especially when deep learning‐based AI algorithms are applied. The successful combination of different brain imaging modalities using deep learning remains a challenging yet crucial research topic. The integration of structural and functional modalities is particularly important for the diagnosis of various brain disorders, where structural information plays a crucial role in diseases such as Alzheimer's, while functional imaging is more critical for disorders such as schizophrenia. However, the combination of functional and structural imaging modalities can provide a more comprehensive diagnosis. In this work, we present MultiViT, a novel diagnostic deep learning model that utilizes vision transformers and cross‐attention mechanisms to effectively fuse information from 3D gray matter maps derived from structural MRI with functional network connectivity matrices obtained from functional MRI using the ICA algorithm. MultiViT achieves an AUC of 0.833, outperforming both our unimodal and multimodal baselines, enabling more accurate classification and diagnosis of schizophrenia. In addition, using vision transformer's unique attentional maps in combination with cross‐attentional mechanisms and brain function information, we identify critical brain regions in 3D gray matter space associated with the characteristics of schizophrenia. Our research not only significantly improves the accuracy of AI‐based automated imaging diagnostics for schizophrenia, but also pioneers a rational and advanced data fusion approach by replacing complex, high‐dimensional fMRI information with functional network connectivity, integrating it with representative structural data from 3D gray matter images, and further providing interpretative biomarker localization in a 3D structural space.

## Graphical abstract

The MultiViT model combines structural and functional neuroimaging data for the prediction of schizophrenia and integrates vision transformers with cross‐attention layers in order to preserve mutual information. The pipeline generates highly interpretable cross‐attention‐based brain saliency maps and emphasizes functional network connectivity patterns related to the disorder.

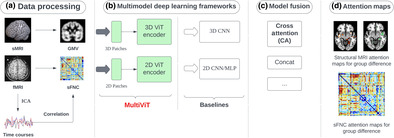

## 1 INTRODUCTION

Deep learning has emerged as a rapidly evolving field in recent years, significantly contributing to addressing various challenges in computer vision and image processing tasks. The success of convolutional neural networks (CNNs) and their numerous adaptations have resulted in the widespread adoption of deep learning techniques in neuroimaging research (Abrol et al., 2021). Numerous studies have employed 3D CNN models to predict intricate brain disorders such as schizophrenia (SZ), with the majority of these studies concentrating on unimodal data (Lin et al., 2022; Oh et al., 2019; Qureshi et al., 2019). The vision transformer (ViT), an adaptation of the transformer model originally developed for natural language processing (Vaswani et al., 2017), has recently emerged as a prominent model in computer vision (Dosovitskiy et al., 2020). Demonstrating potential to outperform traditional CNN models, ViTs are increasingly utilized in various tasks such as image classification (Bazi et al., 2021), image segmentation (Strudel et al., n.d.), and object detection (Beal et al., 2020). ViT can effectively substitute CNN‐based models due to its superior performance on large‐scale image datasets like ImageNet. The self‐attention (SA) mechanism in ViTs results in larger receptive fields and better encoding of spatial relationships among different features, offering a structural advantage over conventional CNN models (Han et al., 2022; Zhao et al., 2020). In the field of medical imaging, researchers are increasingly exploring hybrid CNN‐transformer models (Li et al., 2022; Zhou et al., 2023) due to the scarcity of datasets resulting from privacy concerns. Some researchers are developing more advanced deep learning models based on multimodal image fusion to address the limitations of single modality information and data volume.

Schizophrenia is a highly complex brain disorder characterized by a range of symptoms, including delusions, hallucinations, disorganized thinking, and significant social or occupational dysfunction. Neuroimaging data can illuminate the brain's complex regions and pathways linked with mental disorders such as SZ (Du et al., 2012; Sui et al., 2015). Some studies suggest that SZ is primarily a disorder of functional brain connectivity, and that these irregularities in how brain regions communicate may underlie many of the disorder's characteristic symptoms (Yu et al., 2012; Zhao et al., 2018). For example, functional MRI (fMRI) has been identified as a valuable diagnostic tool, with numerous studies utilizing AI‐assisted diagnostics through fMRI to enhance the detection and understanding of SZ (Ghanbari et al., 2023; Qureshi et al., 2019). In addition, other research suggests that structural changes in the brain, particularly in gray matter volume (GMV) from structural MRI(sMRI), are associated with functional impairments and symptoms observed in SZ (Gupta et al., 2015; Gur et al., 1999; Hulshoff Pol et al., 2002). GMV changes are significant because gray matter includes neuronal cell bodies and synapses, which are critical for processing information in the brain; alterations in these areas can drastically affect cognitive and emotional functions. Thus, assessing GMV can provide essential insights into the neurological underpinnings of SZ. The integration of both structural and functional information, particularly through various medical imaging modalities, is critical to the effective diagnosis of SZ.

In this study, we introduce *MultiViT*, a multimodal interpretable deep learning model that integrates structural and functional neuroimaging data for AI‐assisted diagnosis of SZ. (1) The MultiViT model merges 3D GMV data with lightweight two‐dimensional FNC matrices derived from ICA‐processed fMRI data, effectively combining structural and functional information to significantly improve diagnostic accuracy. (2) The model employs cross‐attention (CA) mechanisms to fuse features processed by ViT encoders from both GMV and FNC data, which has led to a significant increase in model performance, achieving an AUC of 0.833. In addition, *MultiViT* outperformed a number of single‐modality and multimodality baselines composed of different deep learning backbones, benefiting in particular from the computational efficiency of using FNC as a lightweight two‐dimensional matrix compared to higher‐dimensional fMRI data. (3) In addition, we use attention rollout techniques in MultiViT to extract weights from different attentional layers and generate anatomical brain saliency maps based on 3D structural space. These attention maps identify brain regions that serve as biomarkers for SZ. By integrating weights from cross‐attentional layers, the model also pinpoints functional biomarkers, enabling a comprehensive analysis that correlates functional patterns with structural changes.

## 2 RELATED WORKS

Previous neuroimaging research using deep learning architectures, especially ViT, has predominantly focused on unimodal applications. For example, Singla et al. (2022) developed a 3D ViT model for gender prediction using structural MRI data, achieving an AUC of over 0.9 on the ABCD dataset. Nevertheless, the advantages of multimodal approaches over unimodal methods in neuroimaging are increasingly being recognized. For example, Zhou et al. (2023) used a CNN‐transformer mixture to classify brain images across multiple datasets, demonstrating significant effectiveness on Alzheimer's disease datasets. Venugopalan et al. (2021) presented a comprehensive framework that combines MRI, EHR, and SNP data to improve Alzheimer's disease prediction, outperforming single‐modality models.

As the field of multimodal research grows, the application of ViT and other deep learning models for multimodal studies in neuroimaging is gradually increasing. Odusami, Maskeliūnas, and Damaševičius (2023) proposes a multimodal fusion approach using discrete wavelet transform optimized with transfer learning to effectively combine MRI and PET data for early detection of Alzheimer's disease. The architecture fuses structural and functional information at the “pixel level” and improves diagnostic accuracy by integrating data at the individual pixel level in the images. Xing et al. (2022) presents a novel model using ViT applied to 2D fusion images derived from 3D PET scans, demonstrating superior accuracy and AUC values compared to traditional 3D/2D CNN methods when evaluated with ADNI data. Kadri et al. (2023) presents two novel methods for Alzheimer's disease diagnosis using advanced neural architectures that combine elements of CNNs and transformers, demonstrating high classification accuracies on the OASIS dataset. However, the effective design of data fusion modules within deep learning models remains a significant challenge worthy of further exploration in medical imaging. Tang et al. (2022) proposes a multimodal medical image fusion method called MATR, which enhances the global semantic extraction capability through adaptive convolution and adaptive transform to solve the limitations of retaining global context information. However, recent data fusion methods and multimodal deep learning architectures in the medical image field require extensive experiments and training to achieve optimal fusion results, which can be time‐consuming and resource‐intensive.

In addition, researchers are exploring data fusion methods in medical imaging while investigating how to create interpretable deep learning models. Odusami, Maskeliūnas, Damaševičius, and Misra (2023) proposes a methodology for early diagnosis of Alzheimer's disease by combining PET and MRI images through a three‐channel phase feature learning model for early fusion that simultaneously integrates and concatenates neuroimaging data from both modalities; the novelty of the model lies in achieving high specificity and providing interpretability through an explainable artificial intelligence (XAI) model, making the results transparent and understandable. El‐Sappagh et al. (2021) uses explainable AI by providing global and instance‐based explanations of the random forest classifier using the SHapley Additive exPlanations (SHAP) feature attribution framework and implementing various explainers based on decision trees and fuzzy rule‐based systems to help physicians understand the predictions.

## 3 METHODS

**FIGURE 1:**
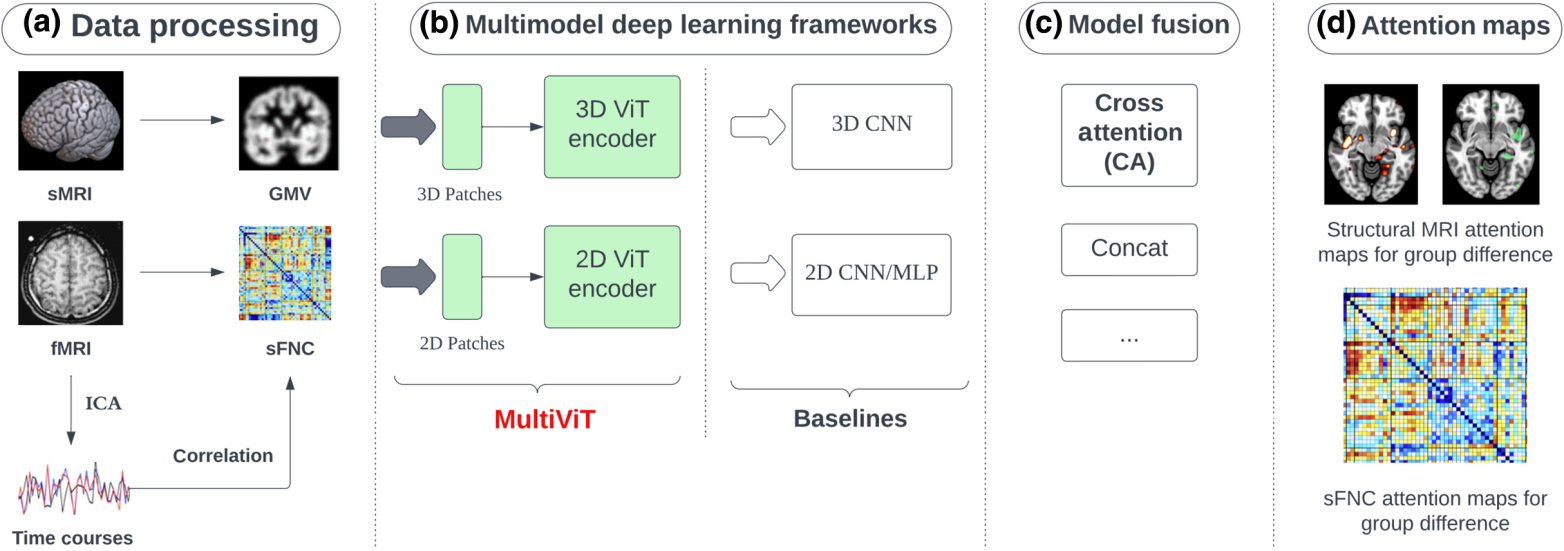
The refined pipeline is as follows: (a) A data pre‐processing module is employed for sMRI segmentation and fMRI pre‐processing. This utilizes the NeuroMark pipeline (Du et al., 2020), to convert the data into FNC and time courses. (b) Several deep learning models have been proposed to handle multimodal data. This includes methods that concatenate 3D and 2D CNN models, serving as our baselines. Notably, our novel model, MultiViT, demonstrates superior performance compared to traditional concatenation approaches using pure CNNs. (c) Various data fusion methodologies were evaluated, encompassing concatenation, weighted MLP, and cross‐attention. Within the scope of MultiViT, the cross‐attention mechanism was selected, given its advantageous impact on model interpretability. (d) Subsequently, 3D and 2D attention maps were formulated by leveraging the weights from the ViT attention layer on both MRIs and FNCs. CNN, convolutional neural network; MLP, multi‐layer perceptron.

Our methodology comprises the following stages: Initially, we conduct pre‐processing on the input data (sMRI and fMRI) utilizing established pipelines. This involves generating 3D gray matter images from sMRI using the unified segmentation model in the SPM toolbox and computing fMRI features employing the NeuroMark pipeline (Du et al., 2020), a fully automated spatially constrained independent component analysis. Subsequently, we construct a 2D static FNC matrix. Secondly, we concurrently analyze the structural and functional MRI modalities employing a multimodal deep learning framework that encodes the data through specialized architectures. We evaluated a range of multimodal pipelines, exploring combinations of different architectures like 3DCNN, 3DViT, 2DCNN, and MLP. Among these, the MultiViT model emerged as a benchmark, outperforming other deep learning and machine learning variants. Finally, we developed an interpretable architecture capable of generating attention maps from ViT encoders for 3D structural MRI and 2D FNC data. These maps can be utilized to identify potential brain areas and functional parts by multimodal MRI data concerning SZ. Figure 1 delineates the general outline of our research.

### 3.1 Data and pre‐processing

In this study, we employed two datasets related to clinical research on SZ, Table 1 shows the subjects' information datasets. The first dataset was compiled from three distinct studies, namely fBIRN (Functional Imaging Biomedical Informatics Research Network) with seven sites, MPRC (Maryland Psychiatric Research Center) with three sites, and COBRE (Center for Biomedical Research Excellence) with one site. This resulted in a total of 827 participants, including 477 control individuals (mean age: 38.76 ± 13.39, 213 females, 264 males) and 350 SZ individuals (mean age: 38.70 ± 13.14, 96 females, 254 males). The fBIRN data was collected using the same parameters for resting‐state fMRI (rsfMRI) at all sites, employing a standard gradient echo‐planar imaging (EPI) sequence with a repetition time (TR) and echo time (TE) of 2000/30 ms, a voxel spacing size of 3.4375 × 3.4375 × 4 mm, and a field of view (FOV) of 220 × 220 mm. The data was collected using six Siemens Tim Trio 3‐Tesla scanners and one General Electric Discovery MR750 3.0 Tesla scanner. For the COBRE data, rsfMRI images were collected using a standard EPI sequence with a TR/TE of 2000/29 ms and a voxel spacing size of 3.75 × 3.75 × 4.5 mm a FOV of 240 × 240 mm, using a 3‐Tesla Siemens Tim Trio scanner. The MPRC data was collected using three different 3‐Tesla Siemens scanners, including the Siemens Allegra, Trio, and Tim Trio (Meng et al., 2022).

**TABLE 1:** Dataset details.

Dataset	Age	Gender (female/male)	Race
Dataset1	TC: 38.76 ± 13.39 SZ: 38.70 ± 13.14	TC: 213/264 (44%) SZ: 96/254 (27%)	No record (from US hospitals)
Dataset2	TC: 29.81 ± 8.68 SZ: 28.98 ± 7.63	TC: 167/159 (51%) SZ: 229/260 (45%)	Chinese ethnic Han (from Chinese hospitals)

The second dataset consisted of 815 participants, collected from seven Chinese hospitals, including Peking University Sixth Hospital, Beijing Huilongguan Hospital, Xinxiang Hospital Simens, Xinxiang Hospital GE, Xijing Hospital, Renmin Hospital of Wuhan University, and Zhumadian Psychiatric (Yan et al., 2019) Hospital. The participants included 326 control individuals (mean age: 29.81 ± 8.68, 167 females, 159 males) and 489 SZ individuals (mean age: 28.98 ± 7.63, 229 females, 260 males), all of whom were Han Chinese. The resting‐state fMRI data were collected using three different 3‐Tesla scanners across the seven sites, including the Siemens Tim Trio, Siemens Verio, and Signa HDx GE Scanner. The participants were instructed to lie still and relax while remaining awake and calm.

The preprocessing of fMRI included slice timing correction, realignment, normalization to the EPI template, and finally smoothing with a 6 mm kernel. Details of preprocessing steps can be found in our previous studies (Du et al., 2020). Moreover, the sFNC data was calculated using cross‐correlation among fMRI time series obtained through independent component analysis (ICA), employing a fully automated spatially constrained ICA algorithm and the neuromark_fMRI_1.0 template as spatial priors. The sMRI data were preprocessed using a voxel‐based morphometry pipeline and modulated by the Jacobian of the spatial transform to produce voxelwise GMV data.

### 3.2 Transformer

The transformer model, introduced by Vaswani et al. (2017), has had a profound impact on the field of NLP and, more broadly, deep learning. This innovative architecture overcomes the limitations of traditional RNNs and CNNs in capturing long‐range dependencies and parallelization. Central to the transformer model is the concept of SA mechanisms, which enable the model to weigh the importance of each token in a sequence while considering the relationships between tokens at various positions. This novel approach has led to significant advancements in tasks such as machine translation, text summarization, and question‐answering. Moreover, the transformer model has served as the foundation for the development of numerous state‐of‐the‐art pre‐trained models, such as BERT, GPT, and T5, which have established new performance benchmarks across a wide range of NLP tasks. Consequently, the transformer model has become an indispensable building block in the realm of deep learning, inspiring new research directions and applications beyond natural language processing.

The ViT represents a notable extension of the transformer model from natural language processing to the domain of computer vision. Introduced by Dosovitskiy et al. (2020), ViT adapts the SA mechanisms of the transformer to process image data by dividing input images into non‐overlapping patches and linearly embedding them into a sequence of tokens. As a result, ViT is capable of capturing intricate spatial relationships and long‐range dependencies within images. This groundbreaking approach has demonstrated exceptional performance on various computer vision tasks, including image classification, object detection, and segmentation, frequently outperforming traditional CNNs. The success of ViT is largely attributable to its ability to utilize large‐scale image datasets for pre‐training, which allows the model to learn more expressive and transferable visual representations. Consequently, the ViT has emerged as a potent and versatile instrument in the field of computer vision, spurring novel research avenues and the development of hybrid models that combine the strengths of transformers and CNNs to address a wide range of visual tasks.

### 3.3 Self‐attention and cross‐attention

The pioneering application of the SA mechanism (Sukhbaatar et al., 2019) was in the NLP domain, where it was utilized to determine the level of emphasis of each word in an input sequence. Subsequently, the ViT models have expanded the use of SA to image embeddings. Given an input image embedding $X\in {ℛ}^{d_{\mathrm{seq}}\times d_{\text{model}}}$, three trainable weight matrices $W^{Q}\in {ℛ}^{d_{\text{model}}\times d_{q}},W^{K}\in {ℛ}^{d_{\text{model}}\times d_{k}}$, and $W^{V}\in {ℛ}^{d_{\text{model}}\times d_{v}}$ are utilized to project X into query matrix $Q\in {ℛ}^{d_{\mathrm{seq}}\times d_{q}}$, key matrix $K\in {ℛ}^{d_{\mathrm{seq}}\times d_{k}}$, and value matrix $V\in {ℛ}^{d_{\mathrm{seq}}\times d_{v}}$.

(1) $$\begin{array}{l} Q={\mathrm{XW}}^{Q}\\ K={\mathrm{XW}}^{K}\\ V={\mathrm{XW}}^{V} \end{array}$$

Then, we have the SA function that calculates the score matrix:

(2) $$\mathrm{SA}\left(Q,K,V\right)=\text{softmax}\left(\frac{{\mathrm{QK}}^{T}}{\sqrt{d_{k}}}\right)V$$

where $d_{q}=d_{k}$, which is the dimension of the query and key matrix. In the ViT models, multi‐head self‐attention (MHSA) separates the original input embeddings into multiple heads, then computes the concatenation from each head's self‐attention result. This can be represented as:

(3) $$\begin{array}{l} {\text{head}}_{i}=\text{Attention}\left({\mathrm{QW}}_{i}^{Q},{\mathrm{KW}}_{i}^{K},{\mathrm{VW}}_{i}^{V}\right)\\ \text{MHSA}\left(Q,K,V\right)=\text{Concat}\left({\text{head}}_{1},\ldots ,{\text{head}}_{i}\right)W^{O} \end{array}$$

where $W_{i}^{Q},W_{i}^{K}$, and $W_{i}^{V}$ are trainable weight matrices that project the original query, key, and value, respectively, into linearly projected versions in each head_*i*_. The trainable weight matrix $W^{O}$ performs a linear projection to the final result after the concatenation of each ${\text{head}}_{i}$.

Cross‐attention (CA) serves as a cornerstone in transformer‐based deep learning models, allowing for seamless integration of disparate input sources. First introduced by Vaswani et al. (2017), CA empowers a transformer to focus on and assign significance to tokens or features from varying inputs, all the while discerning their inter‐relationships. This flexibility enables the model to adeptly merge and analyze mixed data types, from text and audio to images (Wei et al., 2020), as well as data like structural and functional brain images. The strength of CA lies in its ability to foster optimal inter‐modality information sharing (Lee et al., 2018), which culminates in a holistic data interpretation and enhanced predictive prowess. Owing to its effectiveness, CA has become indispensable in crafting top‐tier models across fields such as NLP, computer vision, and biomedical studies (Gheini et al., 2021).

Given image embeddings, X and Y, the concept of cross‐attention can be likened to self‐attention. From embedding X, we derive queries $Q_{X}$ and keys $K_{X}$, while values $V_{Y}$ are derived from embedding Y. The equation can be written as:

(4) $$\mathrm{CA}\left(Q_{X},K_{X},V_{Y}\right)=\text{Softmax}\left(\frac{Q_{X}K_{X}^{⊤}}{\sqrt{d_{k}}}\right)V_{Y}$$

In models like ViTs, multihead cross‐attention (MHCA) frequently features in the design blueprint. Elaborating further, for each head i, we have learned weight matrices $W_{\mathrm{Qi}}^{X}$, $W_{\mathrm{Ki}}^{X}$, and $W_{\mathrm{Vi}}^{Y}$. With these, for every head i, we calculate:

(5) $$\begin{array}{l} Q_{\mathrm{Xi}}=XW_{\mathrm{Qi}}^{X}\\ K_{\mathrm{Xi}}=XW_{\mathrm{Ki}}^{X}\\ V_{\mathrm{Yi}}=YW_{\mathrm{Vi}}^{Y} \end{array}$$

Subsequently, attention is determined for each individual head as:

(6) $${\mathrm{CA}}_{i}\left(Q_{\mathrm{Xi}},K_{\mathrm{Xi}},V_{\mathrm{Yi}}\right)=\text{Softmax}\left(\frac{Q_{\mathrm{Xi}}K_{\mathrm{Xi}}^{⊤}}{\sqrt{d_{k}}}\right)V_{\mathrm{Yi}}$$

Finally, amalgamate the outputs from all heads and apply a transformation using an additional learned weight matrix:

(7) $$\text{MHCA}\left(Q_{X},K_{X},V_{Y}\right)=\left[{\mathrm{CA}}_{1},{\mathrm{CA}}_{2},\ldots ,{\mathrm{CA}}_{h}\right]W_{O}$$

Here, h represents the total number of heads. $W_{O}$ is an output weight matrix, dimensioned as $\left(h\times d_{v},d_{\text{out}}\right)$. In this, $d_{v}$ corresponds to the value vector's dimension, while $d_{\text{out}}$ signifies the anticipated output dimension.

### 3.4 MultiViT

#### 3.4.1 3D ViT pipeline

We describe the 3D ViT model structure of MultiViT, which processes the 3D sMRI input. The input is a 3D sMRI structure with the shape: batch size × 1 × 128 × 128 × 128. This input is segmented into a series of 3D patches with the shape: 1 × 16 × 16 × 16. Each patch is then transformed via linear embedding and position embedding, resulting in an embedding with the shape: *B* × *n* × *d*, where *B* is the batch size, *n* is the number of patches, and *d* is the embedding dimension. After passing through M ViT encoder layers, each incorporating a MHSA mechanism, we obtain a feature representation with the shape: *B* × *n* × *d*, which will be used in the cross‐attention layer.

#### 3.4.2 2D ViT pipeline

We describe the other input branch of MultiViT, which processes the 2D FNC data. The input is a 2D FNC structure with the shape: batch size × 1 × 54 × 54 (after padding). This input is segmented into a series of 2D patches with the shape: 1 × 27 × 27. Each patch is then transformed via linear embedding and position embedding, resulting in an embedding with the shape: *B* × *m* × *d*, where *B* is the batch size, *m* is the number of patches, and *d* is the embedding dimension. After passing through N ViT encoder layers, each incorporating a MHSA mechanism, we obtain a feature representation with the shape: *B* × *m* × *d*, which will be used in the cross‐attention layer.

#### 3.4.3 Data fusion

**FIGURE 2:**
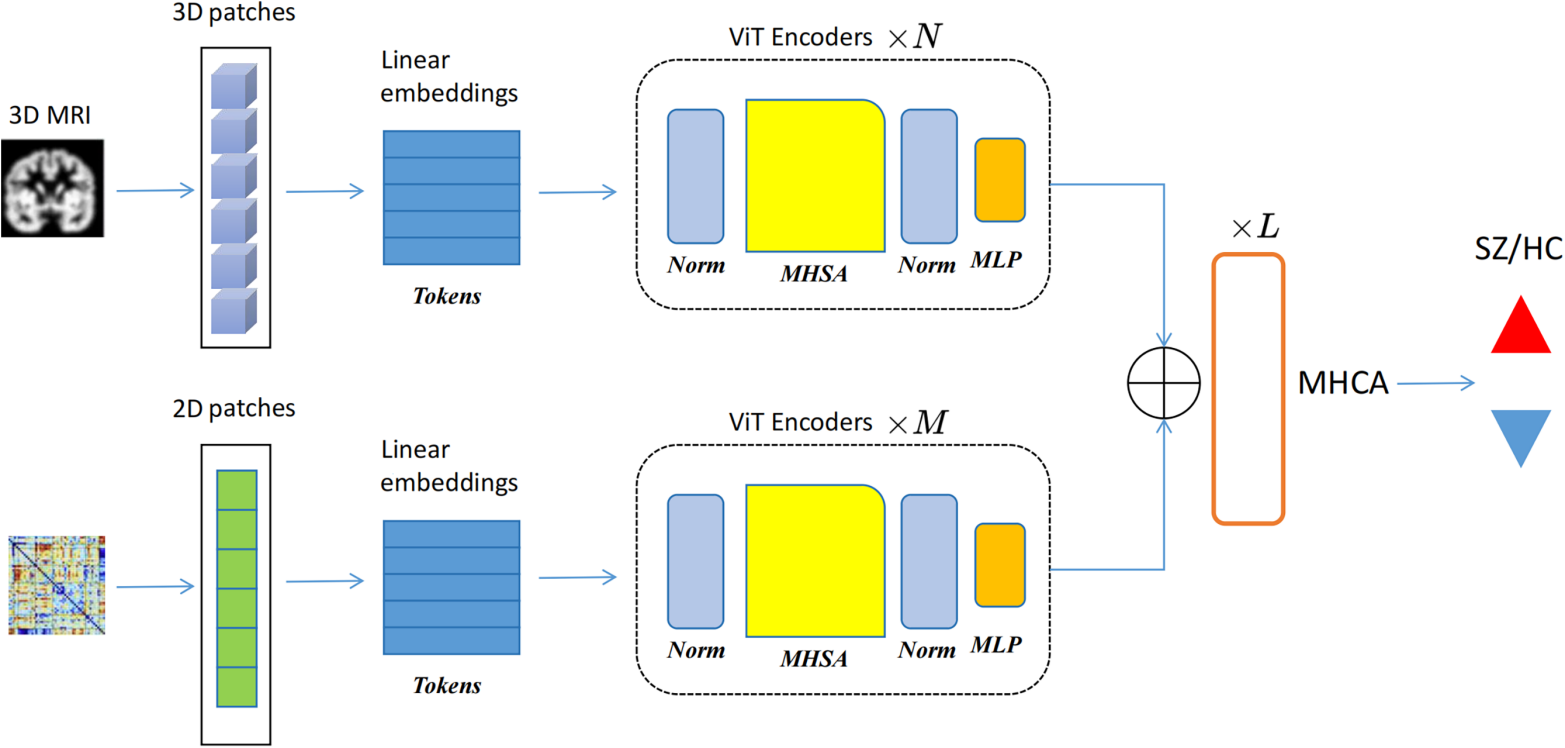
MultiViT: A schematic representation of a multimodal ViT architecture processing MRI data. The process starts by extracting 3D and 2D patches from MRI and FNC data. These patches undergo linear embedding transformations to produce tokens. Subsequently, the tokens are fed into multiple ViT encoders. Within these encoders, the data flows through normalization, MHSA, and MLP blocks. Finally, the combined data is processed by a MHCA module, which classifies the data into two categories: SZ/HC, for schizophrenia and healthy controls, respectively. MHCA, multi‐head cross‐attention; MHSA, multi‐head self‐attention; MLP, multi‐layer perceptron.

We describe the cross‐attention operation that integrates the features obtained from the previous two subsections. The query matrix Q is derived from the 3D sMRI features, while the key and value matrices K and V come from the 2D FNC features. The cross‐attention layer can have multiple heads and multiple layers. The final fused feature representation has the shape: $B\times \left(n+m\right)\times d$. These steps culminate in the final composite representation, which is then passed through an MLP to derive the prediction score. Table 2 and Figure 2 summarize and show the architecture of our MultiViT model.

**TABLE 2:** Summary of MultiViT model pipeline.

	Shape transformation
3D ViT pipeline	
Input	(B, 1, 128, 128, 128)
3D patches	(B, n, 16 × 16 × 16)
Linear embedding + position embedding	(B, n, d)
ViT Encoder	(B, n, d)
2D ViT pipeline	
Input	(B, 1, 54, 54)
2D patches	(B, m, 27 × 27)
Linear embedding + position embedding	(B, m, d)
ViT Encoder	(B, m, d)
Cross‐attention	
Query (3D sMRI features)	(B, n, d)
Key, Value (2D FNC features)	(B, m, d)
Cross‐attention output	(B, n + m, d)
Data fusion	
Concatenation	(B, n + m, d)
MLP	Prediction score

Abbreviations: CNN, convolutional neural network; MLP, multi‐layer perceptron.

### 3.5 Attention map

Our MultiViT model's interpretability is based on using ViT models to obtain attention maps, identifying significant regions and structures within the input sMRI and FNC data. Traditional CNN models can generate saliency maps from input data through gradients, but CNNs focus primarily on local features. This local emphasis often overlooks long‐range dependencies and contextual information essential for a comprehensive understanding of brain patterns.

ViT‐based attention maps offer several advantages over traditional gradient‐based saliency maps from CNN models. The self‐attention mechanisms in ViTs process images holistically, considering the interaction between all components within the image. This global perspective incorporates additional contextual information during the prediction phase, which can enhance precision. Unlike CNNs, which primarily focus on local characteristics, ViTs can capture long‐distance correlations between different image regions. These long‐range dependencies may contain crucial information that traditional models might miss. Furthermore, the interpretability of ViT attention maps surpasses that of CNN saliency maps. By highlighting the regions where the model focuses during prediction, these maps provide insightful perspectives on the structural intricacies of brain function and regions associated with SZ.

Specifically, for 3D sMRI data, our attention maps are realized by mapping attention weights back to the original 3D space. This process results in a 3D weight map that reflects the significance of different brain regions in relation to SZ. We integrate FNC data by extracting attention weights from the CA layers, and the resulting 3D weight map combined with functional information illustrates the correlation between brain structure and SZ after data fusion. This approach provides a precise and comprehensive representation of attention distribution throughout the brain's structure and function. In detail, the attention map A in a ViT model is derived from the attention weights W in the self‐attention layers. For an input image X with patches $X_{1},X_{2},\ldots ,X_{n}$. The final attention map A is then obtained by averaging the attention weights across all heads h and layers l:

(8) $$A=\frac{1}{L}\sum_{l=1}^{L}\frac{1}{H}\sum_{h=1}^{H}W^{\left(l,h\right)}$$

For 3D sMRI data, these attention weights are mapped back to the original 3D space. If X represents the entire 3D MRI, the attention weights W are mapped based on the token positions within this 3D space, resulting in a 3D weight map $A_{3D}$:

(9) $$A_{3D}\left(x,y,z\right)=\sum_{i=1}^{n}W_{i}\cdot \delta \left(x-x_{i},y-y_{i},z-z_{i}\right)$$

where δ is the Dirac delta function, ensuring the weights are applied to the correct spatial locations $\left(x_{i},y_{i},z_{i}\right)$.

When integrating 2D FNC data, the attention weights from the CA layers need to be mapped into the 3D space. These CA layers provide attention weights $W_{\mathrm{CA}}$, which are directly mapped to the 3D MRI space, considering the corresponding spatial coordinates. This results in a 3D weight map from the CA layer attention weights:

(10) $$A_{\mathrm{CA}\_3D}\left(x,y,z\right)=\sum_{i=1}^{n}W_{\mathrm{CA},i}\cdot \delta \left(x-x_{i},y-y_{i},z-z_{i}\right)$$

The combined attention map $A_{\text{comb}}$ is then derived by integrating the 3D sMRI attention map and the 3D‐mapped CA attention map. This integration can be done by averaging or weighted summation, taking into account the contributions from both structural and functional data:

(11) $$A_{\text{comb}}\left(x,y,z\right)=\alpha A_{3D}\left(x,y,z\right)+\beta A_{\mathrm{CA}\_3D}\left(x,y,z\right)$$

where α and β are weighting factors balancing the contributions from structural MRI and functional connectivity data, respectively. This combined 3D weight map provides a good representation of the brain regions associated with SZ, integrating both structural and functional insights.

## 4 EXPERIMENT

### 4.1 Experimental setup

#### 4.1.1 Datasets

In our research, we utilized data from multi‐site studies on SZ, including individuals scanned with a 3 T MRI scanner (*N* = 2130). The sMRI data is three‐dimensional with dimensions of $\left(121,145,121\right)$, and the sFNC data, calculated from 53 regions of interest from fMRI, is two‐dimensional with dimensions of $\left(53,53\right)$. Using Torchio, we resized the sMRI and sFNC data to $\left(120,140,120\right)$ and $\left(54,54\right)$ to fit our model due to the ViT's input characteristics. This open‐source Python library efficiently loads, preprocesses, and augments 3D medical imaging data. Additionally, we augmented our data using various strategies such as RandomCrop, RandomAffine, RandomFlip, and Add GuassianNoise, provided by Torchio.

#### 4.1.2 Models design

To demonstrate the effectiveness of our MultiViT model, we designed and evaluated a series of unimodal and multimodal models. Our experimental design can be divided into three main phases. During the first stage, we focused on unimodal models, which served as our baseline. Since SZ detection can utilize either sMRI data or FNC information, we developed different unimodal models for each type of data. These unimodal representations provided us with essential baseline performance metrics against which we could compare more complex models. In the second phase, we moved to multimodal configurations, combining sMRI and FNC pathways to better capture the multifaceted nature of brain imaging data. We explored basic multimodal models such as the 3D CNN‐CNN and 3DViT‐ViT, which use simple concatenation and weighted MLP techniques for data fusion. These models allowed us to assess the benefits and limitations of simple multimodal integration and provided a benchmark for more sophisticated fusion methods.

The final and most innovative phase of our design involved the MultiViT model. This model uses a ViT‐based architecture that seamlessly integrates both sMRI and FNC data. Specifically, the 3DViT component captures the rich spatial information inherent in sMRI data, while the 2DViT processes FNC data similarly to traditional ViTs. After feature extraction by their respective ViT encoders, the trajectories are fused using a cross‐attention mechanism. This approach enhances the mutual information synergy between the two types of brain imaging data, providing a more sophisticated and effective integration compared to simple concatenation methods.

#### 4.1.3 Training and evaluation

All pipelines, including the baseline models and MultiViT, were trained to utilize advanced techniques such as AdamW optimization, StepLR scheduler, and a 30‐epoch warmup. An 8:1:1 split was employed to select the training, validation, and testing sets. A 10‐fold cross‐validation approach was employed for each model to ensure accurate results. The initial hyperparameters for MultiViT training were set to a learning rate of $3\times 10^{4}$ and a weight decay of $1\times 10^{3}$, totaling 200 epochs. The models were evaluated using general accuracy, balanced accuracy, AUC, F1 score, and precision. The number of parameters for each model is also reported to summarize their complexity. Our experiments used the PyTorch deep learning framework and NVIDIA RTX v100 GPUs.

### 4.2 Results

#### 4.2.1 Model performance

**FIGURE 3:**
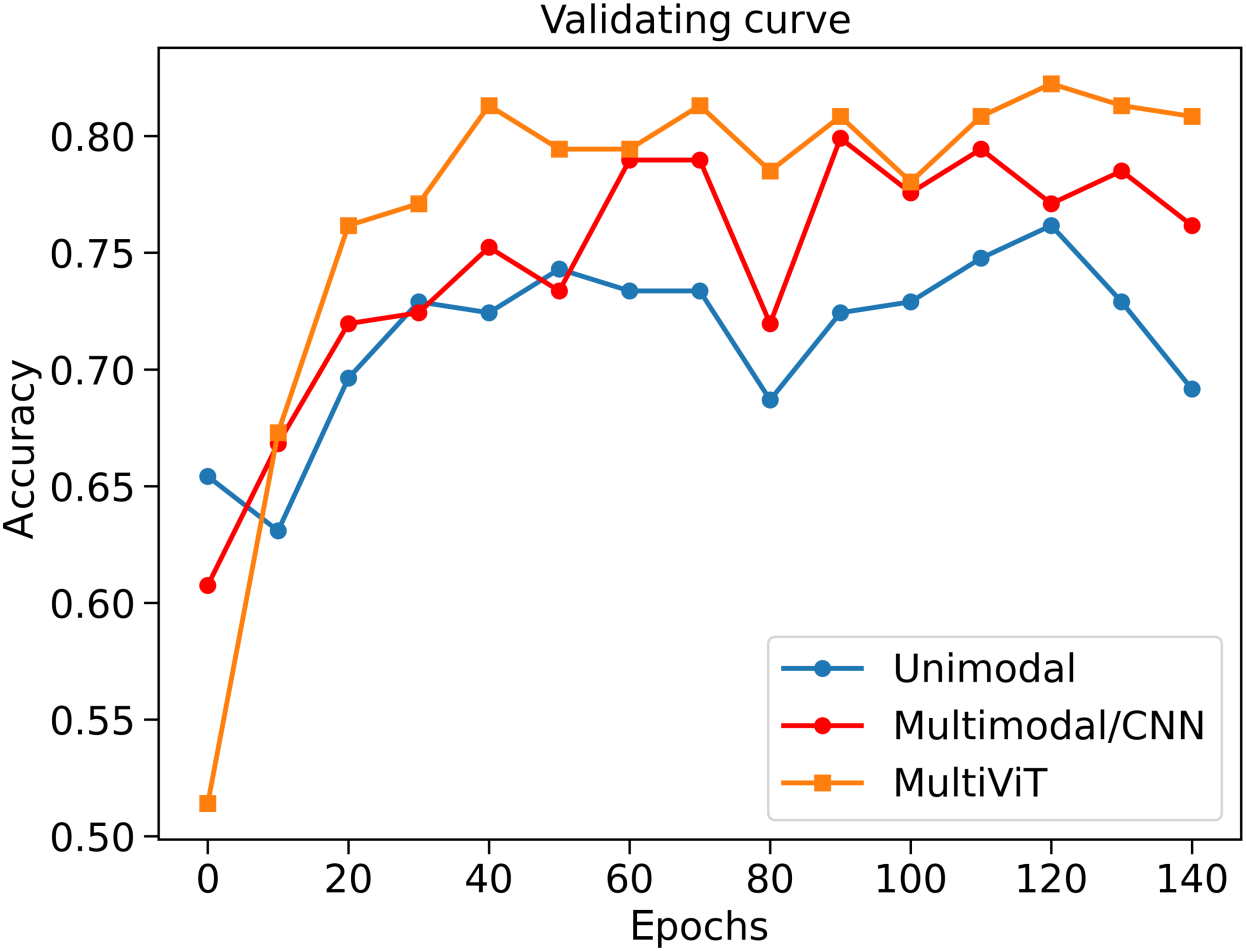
The validation accuracy curves of average(one‐model baselines), average(multimodal baselines), and MultiViT.

In this section, we summarize the model performance based on validation and testing metrics for baselines and MultiViT. Table 3 presents our models' performance, comprising unimodal baselines, multimodal baselines, and MultiViT. The results indicate that MultiViT considerably surpasses other baseline models in terms of balanced accuracy, AUC, and F1 score. Furthermore, while ViT‐based models, including MultiViT, have higher computational costs than CNNs, they offer superior accuracy and overall performance. This trade‐off highlights the robustness and efficacy of the ViT‐based approach despite its computational demands. An important observation from our comparisons is that the cross‐attention mechanism in MultiViT is more effective than pure concatenation and the weighted MLP method. This was evident when comparing MultiViT against other multimodal baselines. Figure 3 shows the validating curve of baseline and MultiViT models.

**TABLE 3:** Testing results of each baseline and MultiViT: we calculate average testing metrics for both unimodal and multimodal baselines, then compare them with our new multimodal pipeline (MultiViT).

Modal	Data	Bal‐Acc	AUC	F1
Unimodal		0.757	0.757	0.747
3DCNN	sMRI	0.775	0.781	0.792
3DViT	sMRI	0.778	0.774	0.784
CrossViT3D	sMRI	0.741	0.743	0.71
MLP	sFNC	0.742	0.741	0.73
ViT	sFNC	0.750	0.745	0.72
Multimodal		0.784	0.780	0.773
3DCNN‐CNN	sMRI + sFNC	0.791	0.784	0.771
3DViT‐ViT	sMRI + sFNC	0.782	0.779	0.774
3DViT‐MLP	sMRI + sFNC	0.780	0.778	0.773
**MultiViT**	**sMRI + sFNC**	**0.831**	**0.833**	**0.840**

### 4.3 Discriminative brain regions discovery

#### 4.3.1 Attention maps for structural imaging

**FIGURE 4:**
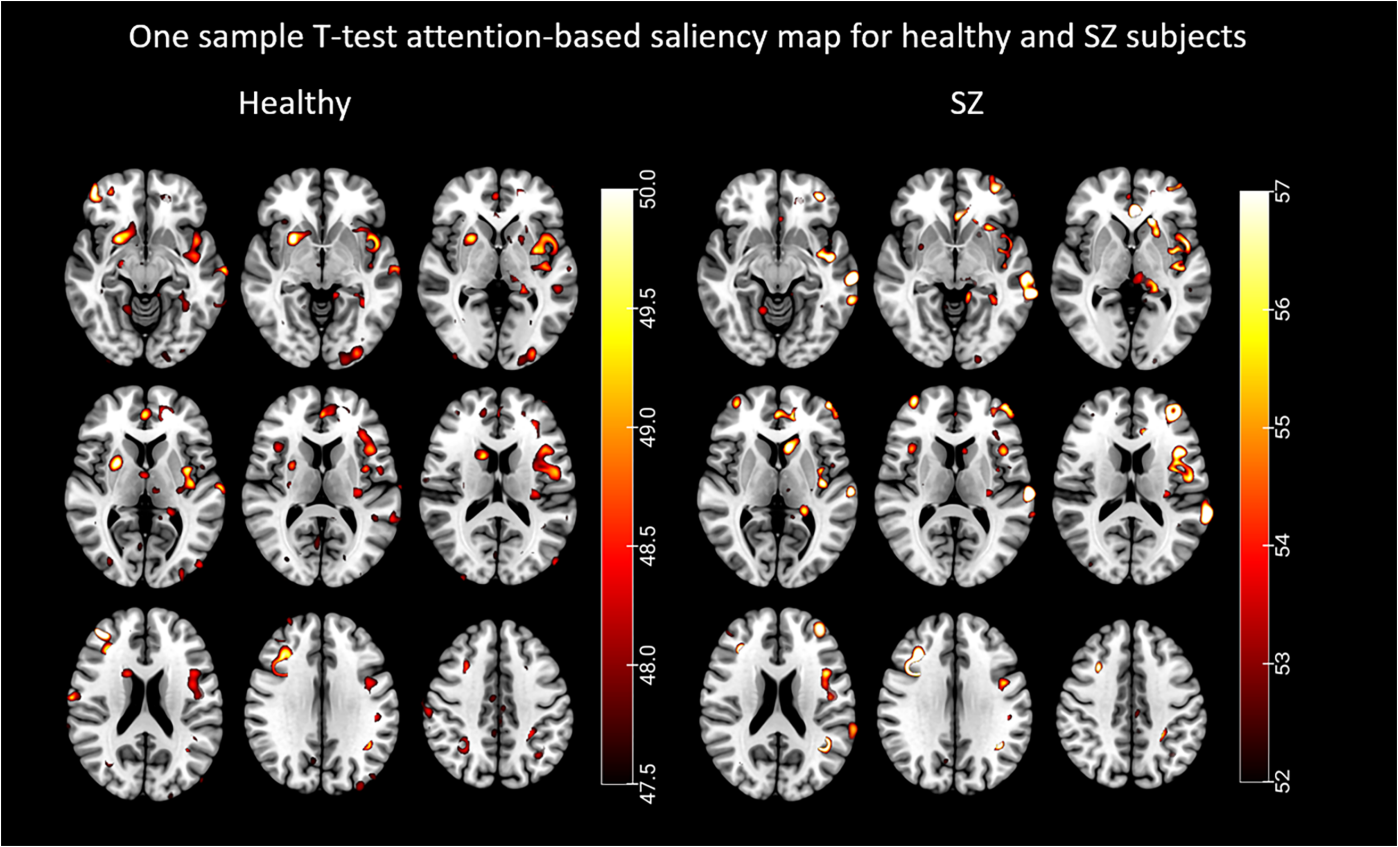
The attention map for one sample *t*‐test shows highlighted brain regions in which the ViT model focused more. On the left are brain regions more relevant to healthy individuals, and on the right are brain regions more relevant to schizophrenia individuals.

**FIGURE 5:**
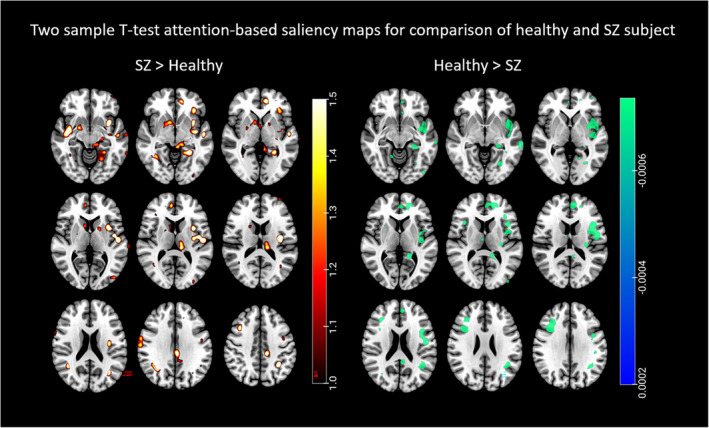
The attention map for the two‐sample *t*‐test with a *p*‐value of 0.02 highlights relevance levels of brain regions associated with healthy individuals and schizophrenia. On the left are brain regions in which schizophrenia is significantly more prevalent than in healthy individuals, indicating that these regions are significantly associated with schizophrenia. Right, are the brain regions more relevant to healthy individuals than schizophrenia patients, that is, these regions have a greater significance in the healthy group.

In this study, using the rollout technique (Touvron et al., 2021), we generated attention‐based saliency maps for individual subjects within the SZ and healthy control (HC) groups. These maps offer a visualization that helps understand specific regions in the brain where the model predominantly focuses. Gaining insights from these attention maps is crucial to discern the model's behavior in its decision‐making process. To analyze the consistency and relevance of these attention patterns, we conducted a one‐sample *t*‐test on the attention values across the voxels. The outcome was an attention map formed of *t*‐values, reflecting the significance of the difference between the observed attention values and a hypothesized population mean for each voxel. We further employed a two‐sample *t*‐test to identify differences in attention patterns between the SZ and HC groups, with the objective of pinpointing brain regions more vulnerable in SZ. Figure 4 visualizes the outcome of the one‐sample *t*‐test, portraying attention patterns for the SZ and HC groups. This map elucidates significant brain regions where the MultiViT model primarily centered its attention, indicating potential structural implications. Figure 5 differentiates brain regions with attention patterns that are more characteristic of SZ versus those typical of HCs.

The one‐sample *t*‐test results indicate that the SZ and healthy groups share major brain areas, which may overlap. The significant brain regions contributing to the MultiViT model for predicting SZ include the anterior cingulum, lingual gyrus, the middle part of the orbital frontal gyrus, precentral gyrus, insula, cerebellum, supplementary motor area, and hippocampus. The brain regions contributing to healthy predictions encompass the caudate nucleus, superior frontal gyrus, fusiform, lingual gyrus, supplementary motor area, the posterior crus II cerebellum, precuneus, precentral gyrus, and superior temporal gyrus. However, the one‐sample *t*‐test cannot distinguish the core regions associated with SZ from those of the healthy group. Consequently, we employed a two‐sample *t*‐test to compare the attention maps of individuals from each testing set between the SZ and healthy groups. The two‐sample *t*‐test results on SZ and healthy groups reveal that some brain regions are more significant in the SZ group than the healthy group, including the left cerebellum, left lingual gyrus, middle temporal gyrus, inferior temporal gyrus, and caudate nucleus. Regions more strongly associated with the healthy group include the left precuneus, right cerebellum, left angular gyrus, right inferior frontal gyrus, left caudate nucleus, and right insula.

#### 4.3.2 Attention maps for functional connectivity

**FIGURE 6:**
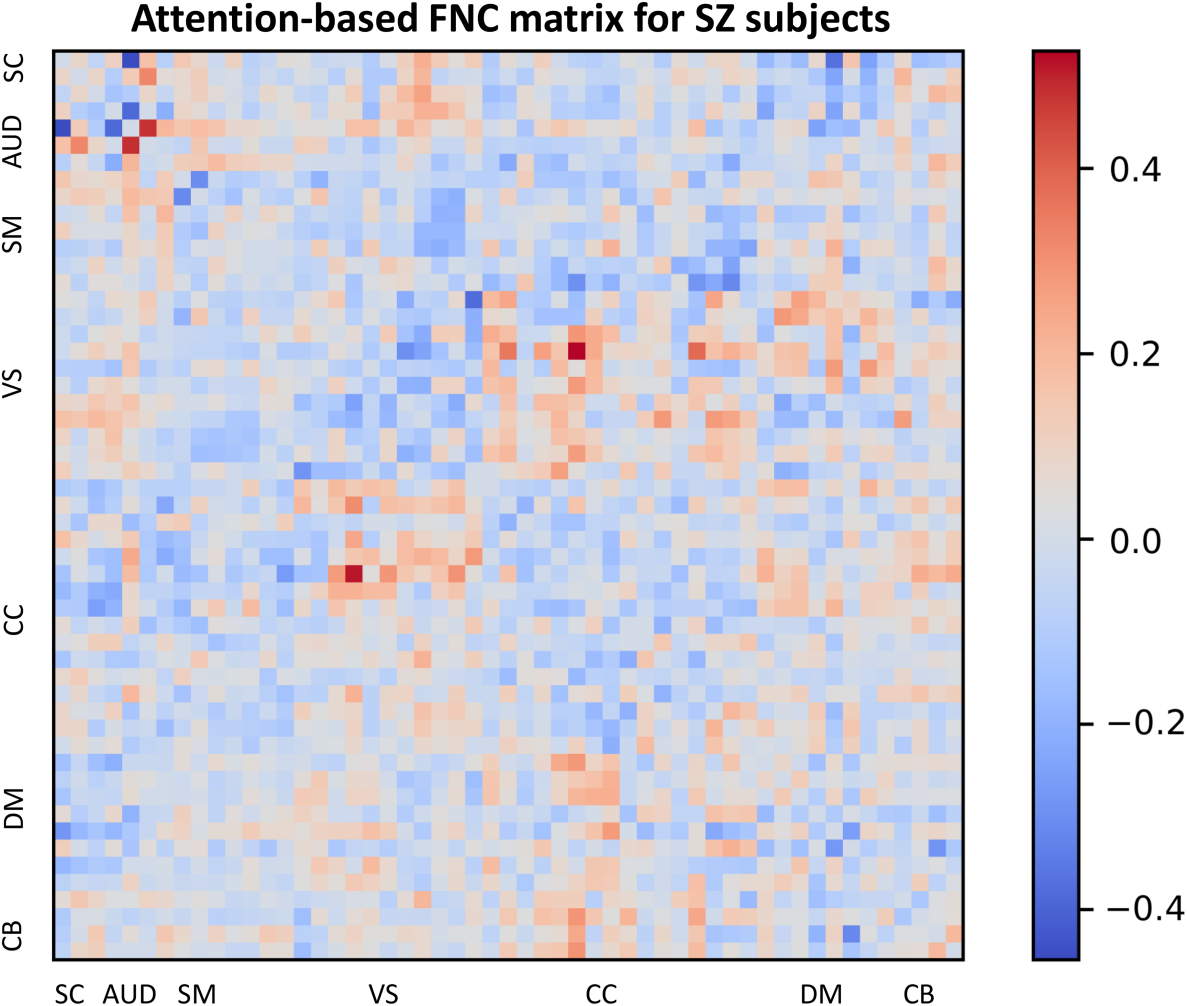
Attention‐based FNC matrix for the schizophrenia patients.

**FIGURE 7:**
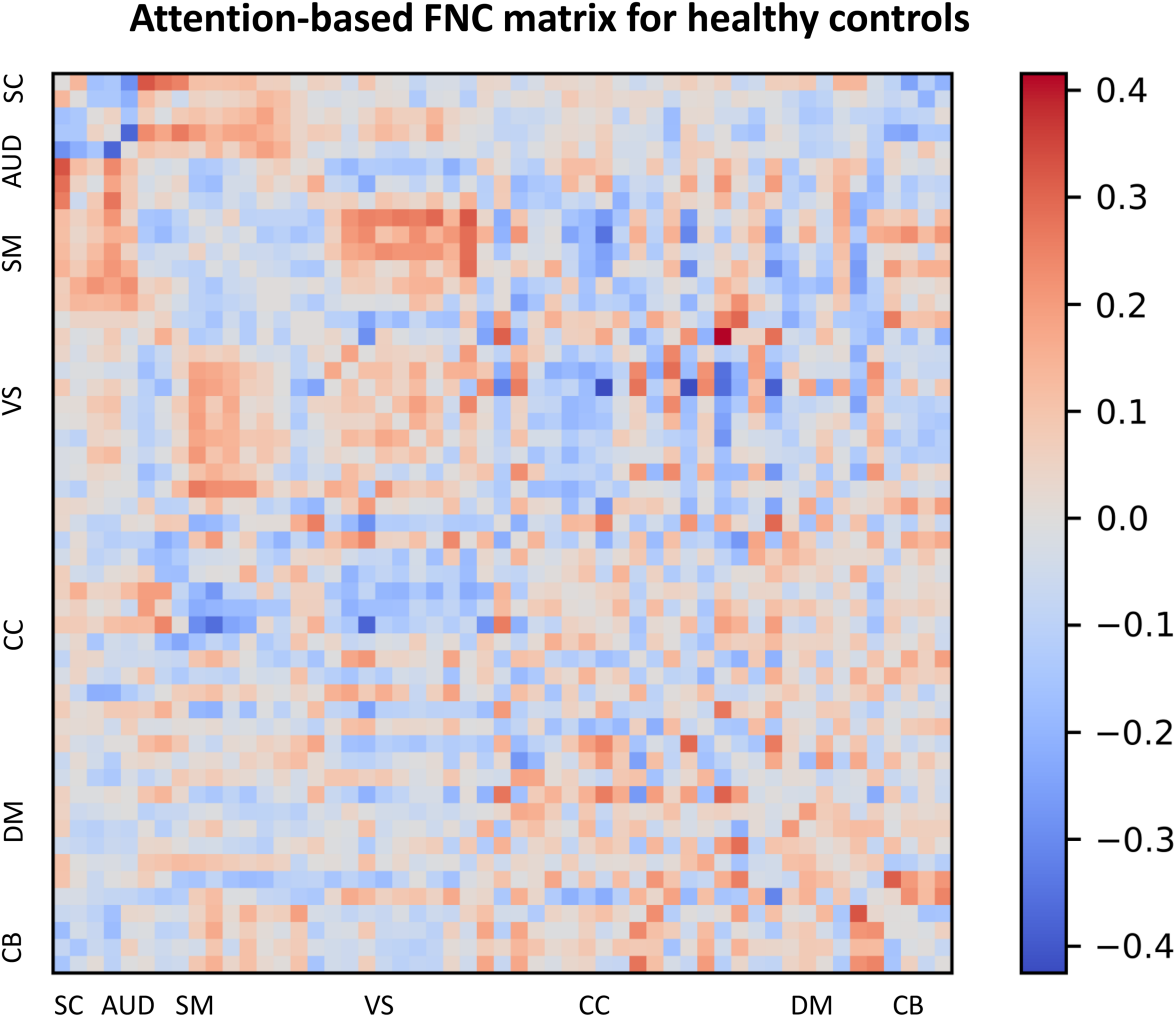
Attention‐based FNC matrix for healthy controls.

**FIGURE 8:**
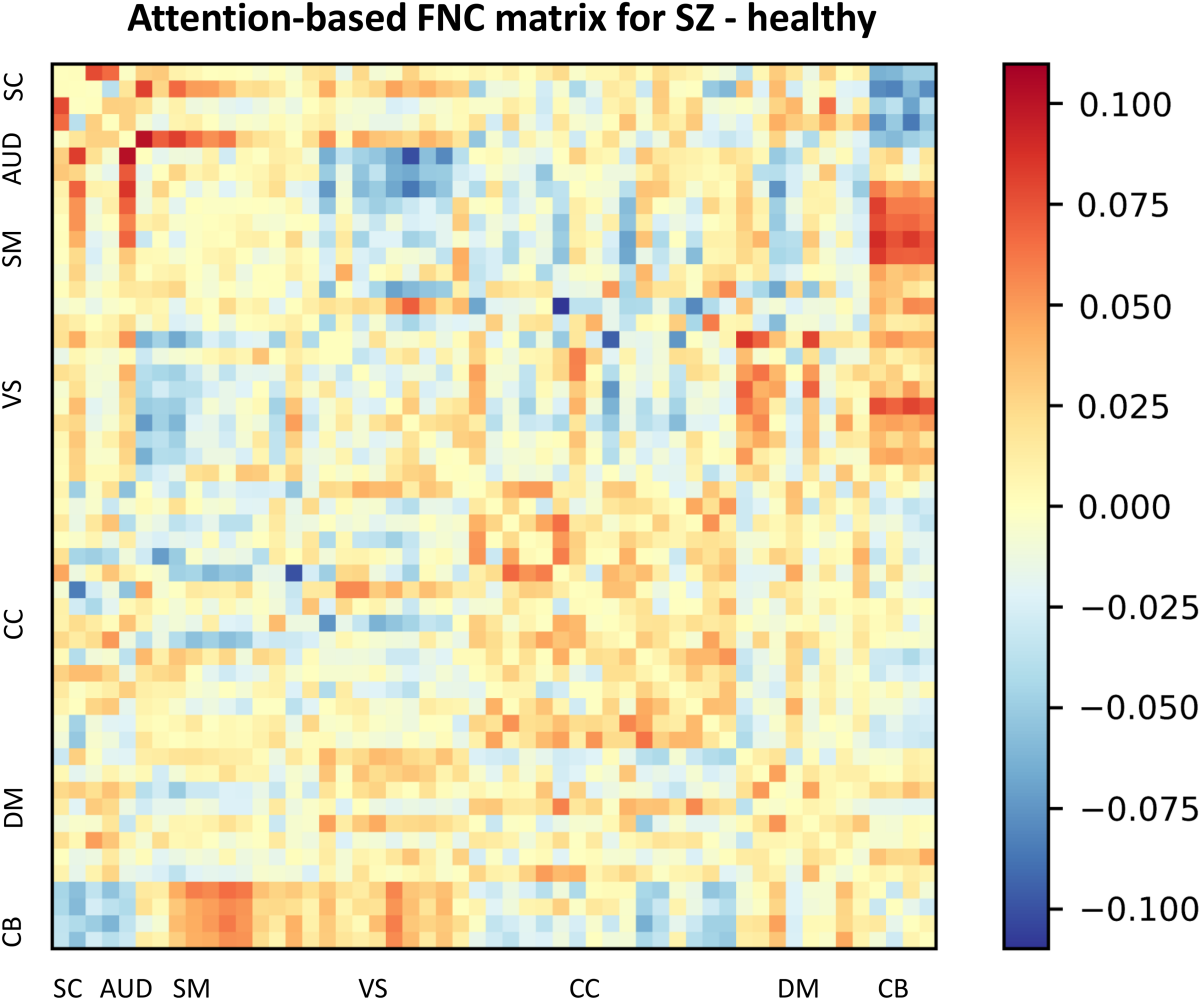
Attention‐based FNC matrix for the difference between schizophrenia over healthy controls.

The FNC matrix represents the temporal correlations among spatially distant neurophysiological phenomena. This matrix is commonly employed to evaluate the associations between disparate brain regions and their interactions during diverse cognitive tasks or in a resting state. In the present investigation, we have incorporated the FNC matrix with attention maps derived from the MultiViT model, facilitating the identification of brain functions that the MultiViT model emphasizes during prediction. These functions may strongly associate with SZ and HC subjects. Figures 6 and 7 illustrate the attention‐based saliency map of the FNC matrix on SZ and HC groups. Nevertheless, discerning the distinctions between SZ and HC subjects within each group remains challenging. To discern the differences between SZ and healthy subjects, attention‐based saliency maps were constructed for the disparities between the SZ and HC groups and the inverse. Figure 8 displays the FNC matrix capable of revealing brain functions predominantly associated with SZ patients rather than HCs. The labels within the FNC matrix denote various brain functions corresponding to distinct connectivities. The regions exhibiting robust connectivities encompass SC‐SM, SC‐VS, CB‐SM, CB‐VS, CC‐CC, and VS‐DM, which could show potential relevant functions related to SZ.

The subcortical (SC) and sensorimotor (SM) interaction plays a pivotal role in subcortical structures, notably the basal ganglia, and thalamus, that are fundamental to the sensorimotor system. The basal ganglia are responsible for initiating and regulating movement by integrating information from the motor cortex and other cortical regions, ultimately promoting seamless and coordinated movement. As a relay center for sensory and motor information, the thalamus facilitates communication with the primary motor and somatosensory cortices to synchronize and modulate motor activity. The SC and ventral stream (VS) interaction primarily involves the processing of visual information and its integration with other cognitive and motor functions. The pulvinar, a subregion of the thalamus, plays a critical role in this interaction as it relays visual information and communicates with various regions of the visual cortex involved in the ventral stream. Interactions between the cerebellum (CB) and the SM network are vital for motor control, coordination, learning, and some cognitive aspects. This interaction ensures precise and well‐coordinated movements and the ability to learn and refine new motor skills. Furthermore, the interaction between the CB and the VS demonstrates visual‐motor integration, wherein the cerebellum can refine and adjust ongoing movements based on visual feedback. Additionally, the cerebellum is involved in visual perception and cognition aspects, such as processing visual motion, estimating time intervals, and predicting future events based on experience. The interaction between different regions of the cingulo‐opercular cortex (CC) can involve integrating various cognitive functions, such as attention, working memory, and error detection. Finally, the interaction between the VS and the dorsal medial prefrontal cortex (DM) involves integrating visual and self‐referential information to support various cognitive processes, such as social cognition, perspective‐taking, and theory of mind.

The subcortical structures (SC) and CB interaction plays an essential role in integrating sensory, motor, and cognitive information, which supports motor control, balance, and cognitive processing. This interaction enables the accurate and coordinated execution of movements, balance and posture maintenance, and sensory and cognitive information processing. In the context of SZ, studies have demonstrated impaired connectivity within the brain, especially within connector hubs, including those in the cerebellum and subcortical regions. This impairment has been found to affect subcortical and cerebellar regions and the regions involved in visual and sensorimotor processing (Yamamoto et al., 2022). The interaction between the VS and the SM network is crucial for integrating visual and motor information to support perception, action, and cognition. This interaction enables the accurate and coordinated execution of movements, the integration of sensory and visual feedback, and the control of attention and working memory. In the case of SZ, functional disintegration between sensory and cognitive processes has been observed. This disintegration is evident in alterations of both amplitude and connectivity within sensory networks, including within‐sensorimotor and sensorimotor‐thalamic connections. Sensory nodes also display widespread alterations in the connectivity with higher‐order nodes (Kaufmann et al., 2015). Finally, the interaction between the VS and the CC integrates visual and cognitive information to support attention, decision‐making, and cognitive control. This interaction enables the efficient and effective allocation of attention, the integration of reward‐related information with decision‐making processes, and the flexible adjustment of cognitive processes in response to changing task demands. The connectivity within these regions is also affected in individuals with SZ, characterized by hypoconnectivity between cingulo‐opercular regions and hyperconnectivity between the thalamus and sensory cortices. These altered connectivity patterns in SZ highlight the need for a comprehensive, data‐driven approach to understand the complex neuropathology of this disorder (Culbreth et al., 2021).

## 5 DISCUSSION AND CONCLUSION

In this investigation, we presented and employed an efficient and comprehensive study on interpretable multimodal ViT‐based model for SZ diagnosis and biomarker identification based on attention maps. This not only provides dependable forecasts for SZ using clinical data, but also discerns connections between dual modalities. Our experiments leveraging FNC data as a computational substrate for a deep learning framework yielded results akin to those obtained with fMRI data post‐intensive training (Cai et al., 2020; Steardo et al., 2020). The literature corroborates the idea that structural and functional changes in SZ have been examined in‐depth. Karlsgodt et al. (2010) assessed structural MRI alongside diffusion tensor imaging, presenting evidence that patients with SZ manifest diminished GMV in areas such as the medial temporal, superior temporal, and prefrontal regions, a conclusion resonating with our own observations. Parallelly, they identified functional shifts in SZ, emphasizing pronounced impairments in short‐term memory and decision‐making faculties. DeLisi et al. (2022) amalgamated diverse SZ‐related findings, spotlighting regions like the cerebellum and superior temporal gyrus.

In the field of computer vision, our proposed MultiViT model successfully integrates multimodal data based on different types of brain data, including a 3D high‐dimensional sMRI and a FNC matrix representing brain functional connectivity. This data fusion mechanism ultimately achieves significantly improved SZ detection results compared to single‐modal models. In addition, we employed a novel cross‐attention mechanism to effectively merge feature vectors processed by different ViT encoders. This innovative approach allows for a more comprehensive interpretation of the multimodal data, leading to improved diagnostic accuracy and a deeper understanding of the interplay between structural and functional brain changes associated with SZ. By leveraging the strengths of each modality and the interpretability of attention maps, our model not only provides reliable predictions, but also facilitates the identification of critical biomarkers. This contributes to a better understanding of the pathophysiology of SZ and highlights the potential of multimodal deep learning approaches in medical diagnostics.

However, there is still significant room for improvement in diagnosing SZ using deep learning techniques alongside structural and functional MRI data. For example, our work falls short in terms of interpretability. While simple attention maps are considered in some studies to reflect the model's “attention” to the data, there is significant potential for improvement. This includes the use of more advanced attention mechanisms or updated attention maps, as suggested by other research. Second, our study is limited by the relatively small data set. Therefore, the use of transfer learning methods could further improve detection accuracy. For example, we could use representative learning to train feature extractors for high‐dimensional sMRI data on large datasets, and then fine‐tune them with a smaller set of SZ‐related sMRI data. This approach may yield more meaningful results. In addition, representative learning can produce lower‐dimensional features that can further speed up the training and inference processes. By addressing these areas, we can make significant progress in improving the diagnostic capabilities and robustness of our MultiViT model, paving the way for more effective and reliable SZ diagnosis using multimodal deep learning approaches.

In addition, we re‐evaluated the clinical application prospects of our study. In recent years, research on AI‐based diagnostic models, especially for brain diseases, has become quite popular. However, in terms of clinical application, some studies, including our MultiViT model, are still far from real‐world implementation. For clinical diagnosis, it is crucial not only to consider the overall accuracy, but also to minimize the potential negative impact of misdiagnosis. In addition, as an adjunct diagnostic tool, the model should be able to interact with healthcare professionals. Clinicians should be able to adjust the parameters of the model to provide statistical predictions rather than simple binary results. In addition, improving computational efficiency and model generalization is critical for clinical application. An effective clinical diagnostic model should have strong generalizability, meaning that it can provide accurate predictions across diverse patient data. This is another critical area that requires further research. By addressing these considerations, our model will increase its utility as a reliable and interactive tool for healthcare professionals, ultimately contributing to better patient outcomes in the diagnosis and treatment of SZ.

## CONFLICT OF INTEREST STATEMENT

None of the authors has any conflicts of interest to declare in relation to this manuscript. This includes any financial, personal, or professional interests that could be construed to influence the work reported in this paper. We confirm that the content of the manuscript has not been influenced by any external interests, and all research was conducted in accordance with ethical standards. This statement is true to the best of our knowledge and belief, and any potential conflicts will be disclosed promptly should they arise in the future.

## Supporting information


**Appendix S1:** Supporting information.

## Data Availability

The data that support the findings of this study are available from the corresponding author upon reasonable request.
