# Supplementary material for: A multimodal vision transformer for interpretable fusion of functional and structural neuroimaging data
Source: Hum Brain Mapp. 2024 Nov 26;45(17):e26783. doi: 10.1002/hbm.26783 (PMC11599617; doi:10.1002/hbm.26783)
Supplement: Supplementary file 1 — Appendix S1: Supporting information. [file HBM-45-e26783-s001.pdf]

Data and Code Availability Statement:

The data and code used in this study are currently confidential and not available for public access. We acknowledge the importance of data and code sharing in scientific research for validation and replication purposes. Therefore, we are considering options for future release. Our decision to maintain confidentiality at this stage is guided by ongoing research considerations and proprietary interests. We intend to review this decision in due course and will provide updates regarding the availability of our data and code as appropriate. Further inquiries about our data and code can be directed to the corresponding author.

Yuda Bi
Anees Abrol
Zening Fu
Vince Calhoun

Tri-institutional Center for Translational Research in Neuroimaging and Data Science (TReNDS),
{ybi3, vcalhoun}@gsu.edu
